# Effect of denture and soft tissue disinfection on edentulous biofilms

**DOI:** 10.1128/spectrum.01608-26

**Published:** 2026-09-23

**Authors:** Amit Sachdeo, Yash Brahmbhatt, Mabi Singh, Athena Papas

**Affiliations:** 1 Department of Prosthodontics, Tufts University School of Dental Medicine 52291, Boston, Massachusetts, USA; 2 Tufts University School of Dental Medicine, Boston, Massachusetts, USA; 3 Division of Oral Medicine, Department of Diagnostic Sciences, Tufts University School of Dental Medicine 52291, Boston, Massachusetts, USA; 4 Department of Basic and Clinical Translational Sciences, Tufts University School of Dental Medicine 52291, Boston, Massachusetts, USA; University of the Pacific Arthur A Dugoni School of Dentistry, San Francisco, California, USA

**Keywords:** oral biofilm, denture disinfection, soft tissue disinfection, chlorhexidine, edentulous patients, periodontal pathogens, complete dentures, oral microbiota

## Abstract

Oral biofilms play a central role in oral and systemic diseases. Periodontal pathogens such as *Porphyromonas gingivalis* and *Aggregatibacter actinomycetemcomitans* have been detected on dentures and oral mucosa, suggesting denture wearers remain at risk. This study aimed to evaluate whether combined denture and soft tissue disinfection reduces microbial biofilm more effectively than denture cleaning alone. Sixty-one complete-denture wearers were randomly assigned to a test group (*n* = 31; denture disinfection plus soft tissue chlorhexidine treatment) or a control group (*n* = 30; denture disinfection only). Biofilm samples from dentures, soft tissues, and saliva were collected before and after treatment and analyzed for 41 bacterial species using checkerboard DNA–DNA hybridization. Both protocols significantly reduced overall bacterial counts, including pathogens of the red complex and *Streptococcus mutans*. Additional soft tissue disinfection yielded greater reductions on the tongue dorsum but had limited effects at other sites. Professional denture disinfection effectively lowers microbial load in edentulous patients, and adjunctive soft tissue treatment provides added benefit. Regular implementation of this protocol may enhance oral hygiene and contribute to improved systemic health outcomes in the denture-wearing population.

**IMPORTANCE:**

Complete dentures can harbor harmful bacteria not only on their dentures but also on oral soft tissues, which may contribute to oral inflammation and potentially affect systemic health. This study demonstrates that professional denture disinfection substantially reduces microbial biofilm, while the addition of soft tissue disinfection provides further improvement, particularly on the tongue. These findings support a comprehensive approach to denture hygiene that addresses both the prosthesis and the soft tissues rather than the denture alone. Incorporating combined disinfection into routine care may help improve oral hygiene, reduce the burden of disease-associated bacteria, and promote better long-term oral and systemic health in edentulous patients.

## INTRODUCTION

Bacteria in nature usually exist in the form of biofilms; the dense accumulation of bacteria was first reported by Black in 1898 (1–3). Biofilms are an accumulation of bacteria, fungi, or protozoa on solid surfaces and can be found in many places in the environment. In the human body, they can be found adhered to tissue cells, teeth, and artificial implants. According to the United States National Institutes of Health, over 80% of microbial infections in the body are caused by bacteria growing as biofilms (4).

Dental plaque is a classic example of a biofilm in which oral bacteria adhere to the tooth surface and to one another in quite specific patterns (5, 6) and are involved in the pathogenesis of caries (7), periodontitis (8–10), dental implant failures (11–13), denture stomatitis, and oral yeast infections such as candidiasis (14, 15).

Over the years, many researchers have examined the composition and development of oral biofilms in the dentate population (16–22). They found a strong association between *Bacteroides forsythus*, *Porphyromonas gingivalis*, *Treponema denticola*, and *Aggregatibacter actinomycetemcomitans* with clinical measures of periodontal disease, particularly pocket depth and bleeding on probing. It has been reported extensively in the literature that the presence of these periodontal pathogens also has a strong association with various systemic diseases (23–28). Connections with pre-term births, low birth weight, atherosclerosis, diabetes, and risk factors associated with cardiovascular disease and stroke (23–29) have already been made. Periodontal disease was also shown to be a clear risk factor for chronic kidney disease (30). It is therefore clear that there seems to be a strong association between periodontal pathogens and various systemic diseases in the dentate.

For the edentulous, however, literature suggests that *A. actinomycetemcomitans* and *P. gingivalis* disappeared from the oral cavity after extraction of all natural teeth and did not reappear if hard surfaces, in the form of complete dentures, were provided (31–33). One could therefore conclude from those studies that if there was an association between these periodontal pathogens and systemic diseases, then the denture-wearing population was not at a great risk. This information becomes extremely significant with the recent finding of *A. actinomycetemcomitans* and *P. gingivalis* in the complete-denture-wearing population (34). The study by Sachdeo et al. provided the first step in defining the organisms that are associated with the edentulous on both the soft (mucosa) and hard surfaces (denture), and the results clearly show the presence of periodontal pathogens in biofilm samples from the edentulous patients (34). The findings by Sachdeo et al. were later confirmed in a cross-sectional study by Cortelli et al. (35), who also found these periodontal pathogens in the oral mucous membranes of elderly individuals with no teeth.

The presence of *A. actinomycetemcomitans* and *P. gingivalis* in the denture-wearing population (34–37) is of great concern because if there is an association between these microbes and systemic health, as current literature suggests (23–29), then the edentulous population is at equal risk, if not higher, than their dentate counterparts. It therefore becomes extremely important to find a way to eliminate or minimize the presence of these periodontal pathogens from the oral soft tissue surfaces and dentures of such patients.

Several researchers have examined the denture hygiene habits of the edentulous population (38–40). Dikbas et al. (38) surveyed 234 denture-wearing patients on their denture cleaning habits and found that 82.9% of patients claimed their dentists never informed them how to clean their dentures, and only 11.9% of patients were found to have clean dentures. They concluded that most denture wearers did not clean their dentures satisfactorily (38). Similar findings were observed by Marchini et al. (39), who clinically examined 236 complete denture wearers and found that 77.5% of the subjects claimed to have never been given instructions regarding the hygiene of their dentures. They found that self-reported lack of guidance to oral care was statistically related to inflammatory oral conditions (38–40).

In order to disinfect acrylic removable dentures, various methods have also been tried, such as sodium hypochlorite, glutaraldehyde, chlorhexidine, vinegar, and sodium perborate (41–43). Researchers have even microwaved acrylic dentures to see the effectiveness in the reduction/elimination of certain microbes (44, 45).

Overall, the purpose of this investigation was twofold: the first was to develop a method to help reduce microbial biofilm from the oral cavity of the complete-denture-wearing subject, and the second was to see if disinfection of the soft tissues combined with disinfecting the complete dentures had a better outcome at microbial elimination than disinfecting the dentures alone.

## MATERIALS AND METHODS

### Subject population

The subject population consisted of 61 edentulous subjects who used complete maxillary and mandibular dentures daily. The baseline characteristics of these subjects are presented in Table 1. A total of 54% of the subjects were male, and 46% of the subjects were female. Subjects of any racial/ethnic group were accepted for the study if they were in good general health and over 20 years of age, had been edentulous for at least one year, and wore complete maxillary and mandibular dentures daily. Subjects who had had antibiotic therapy in the 3 months before the start of the study or any oral lesions (e.g., candidiasis, ulcerations, leukoplakia, and oral cancer) or systemic condition that required antibiotic coverage for routine dental procedures (e.g., heart conditions, joint replacements, etc.) were excluded from the study.

**TABLE 1:** Demographic features of the subjects

Demographic characteristic	Value
Mean age in years (± SD)	59.6 (± 11.3)
% Males (*N*)^*a*^	54 (33)
% Females (*N*)^*a*^	46 (28)
% Current smokers (*N*)^*a*^	43 (26)
% White^*b*^ (*N*)^*a*^	64 (39)
% African American (*N*)^*a*^	34 (21)
% Asian (*N*)^*a*^	2 (1)

^*a*^ *N* denotes the actual number of subjects.

^*b*^ One subject in the White group reported being Hispanic.

### Treatment

The subject population was divided into two groups for the study: a test group and a control group. A total of 31 subjects were randomly selected for the test group, who received both soft tissue cleaning and denture disinfection, while the remaining 30 patients who served as the control group only received denture disinfection without soft tissue cleaning.

### Test group

Thirty-one randomly selected subjects received soft tissue cleaning and denture disinfection. The dentures were removed from the patients’ mouths and placed in an ultrasonic cleaner (46) for 10 min, containing undiluted tartar and stain-removing solution with type 4 4-sulfamic acid (Patterson Brand Tartar and Stain Remover) (47). While the dentures were being cleaned in the ultrasonic bath, a toothbrush soaked in 20 mL of 0.12% chlorhexidine (Zila Pharmaceuticals, Phoenix, Arizona) was used to brush the dorsum of the tongue for 1 min. A sterile gauze was then soaked with chlorhexidine, and the soft tissues were wiped with the saturated chlorhexidine gauze. If deposits remained on the dentures after 10 min in the ultrasonic bath, the dentures were re-cleaned for 5 min in the ultrasonic cleaner with the same tartar and stain-removing solution. The clinician occasionally had to use a periodontal scaler to gently remove tenacious deposits. The dentures were then brushed and rinsed enough so that the subject could not taste any solution when the dentures were placed back in the mouth. The clinician then poured 20 mL of chlorhexidine into a new cup, and each subject first rinsed with the 20 mL of chlorhexidine for 2 min and then gargled before spitting the chlorhexidine out.

### Control group

Thirty subjects, who served as the control group, did not receive any soft tissue disinfection. However, their dentures were cleaned as described above. Biofilm samples were taken from each of the 61 subjects before and after treatment for all the intra-oral locations as follows.

### Soft tissue samples

Microbial samples were taken from eight separate oral soft tissue locations in each subject using MasterAmp buccal swab brushes (Epicenter Technologies, Madison, Wisconsin, USA) at baseline and immediately after treatment. A total of 488 samples were obtained. The soft tissue samples were obtained by gently stroking each site in an area large enough to yield enough microorganisms for DNA probe analysis. The samples were collected from three areas of the tongue, where one sample was taken from the dorsum of the tongue, one sample from the ventral surface, and one sample from both the lateral surfaces of the tongue. This was done by first taking a sample from the left lateral surface of the tongue and then using the same swab to take a sample from the right lateral surface. Similarly, there was one sample for both the left and right buccal mucosa and the maxillary and mandibular labial vestibules, respectively. Microbial samples were also taken from the floor of the mouth, hard palate, and the maxillary anterior “attached gingiva” (fixed keratinized tissue). The samples were placed in separate tubes containing 0.15 mL Tris EDTA (TE) buffer (10 mM Tris-HCl, 0.1 mM EDTA, pH 7.6), and 0.15 mL 0.5 M NaOH was added.

### Denture samples

Individual plaque samples were taken from the mesio-buccal aspect of the 28 denture teeth at baseline and immediately after cleaning. Thus, 28 samples per subject were taken at two visits (baseline and immediately after denture cleaning and re-insertion in the mouth) for a total of up to 3,416 samples (61 subjects × 28 teeth × 2 visits). Each sample was obtained using separate sterile curettes from the mesio-buccal surface of every denture tooth. The samples were placed in separate tubes containing 0.15 mL of TE buffer and processed as described for the soft tissue samples. An additional sample from the midpoints of the exterior, polished surfaces of each denture’s hard palate was taken using a MasterAmp buccal swab brush. All the dentures had polished exterior surfaces without any rugae.

### Saliva samples

Each subject provided a sample of whole unstimulated saliva by expectorating into a sterile tube before and immediately after treatment. A total of 122 samples were collected. A 0.2 mL sample of whole saliva was mixed with 0.15 mL sterile, filtered TE buffer. A 0.2 mL sample of this mixture was transferred to a new tube, and 0.15 mL of 0.5 M NaOH was added. The samples were processed as described for the soft tissue and denture samples.

*Microbial analysis of the samples*: All samples were analyzed using checkerboard DNA–DNA hybridization to determine the levels of 41 bacterial species presented in Table 2 (48). This technique is used to determine sequence similarity between DNAs of different origins and the amount of sequence repetition within one DNA. It is a useful tool for the enumeration of bacterial species in large samples of microbiologically complex systems (16, 48, 49).

**TABLE 2:** Species for which DNA probes were prepared for the study^*a*^

Species	DNA probes
*Actinobacillus actinomycetemcomitans* 43718 and 29523	*Actinomyces israelii* 12102
*Actinomyces gerencseriae* 23840	*Actinomyces odontolyticus* 17929
*Actinomyces naeslundii genospecies* 1 12104	*Campylobacter gracilis* 33236
*Actinomyces naeslundi genospecies* 2 43146	*Campylobacter showae* 51146
*Campylobacter rectus* 33238	*Capnocytophaga ochracea* 33596
*Capnocytophaga gingivalis* 33624	*Eikenella corrodens* 23834
*Capnocytophaga sputigena* 33612	*Eubacterium saburreum* 33271
*Eubacterium nodatum* 33099	*Fusobacterium nucleatum ss polymorphum* 10953
*Fusobacterium nucleatum ss nucleatum* 25586	*Fusobacterium periodonticum* 33693
*Fusobacterium nucleatum ss vincentii* 49256	*Leptotrichia buccalis* 14201
*Gemella morbilliorum* 27824	*Peptostreptococcus micros* 33270
*Neisseria mucosa* 19696	*Prevotella intermedia* 25611
*Porphyromonas gingivalis* 33277	*Prevotella nigrescens* 33563
*Prevotella melaninogenica* 25845	*Selenomonas noxia* 43541
*Propionibacterium acnes* 11827 and 11828	*Streptococcus constellatus* 27823
*Streptococcus anginosus* 33397	*Streptococcus intermedius* 27335
*Streptococcus gordonii* 10558	*Streptococcus mutans* 25175
*Streptococcus mitis* 49456	*Streptococcus sanguinis* 10556
*Streptococcus oralis* 35037	*Treponema denticola B1*
*Tannerella forsythia* 43037	*Veillonella parvula* 10790
*Treponema socranskii S1*	

^*a*^ All strains were obtained from the American Type Culture Collection (ATCC) except *Treponema denticola* B1 and *Treponema socranskii* S1, which were obtained from Forsyth Dental Center.

In brief, the samples were placed in separate Eppendorf tubes containing 0.15 mL TE (10 mM Tris-HCl, 1 mM EDTA, pH 7.6), and 0.15 mL of 0.5 M NaOH was added to each tube. The samples were lysed, and the DNA was placed in lanes on nylon membranes using a Minislot device (Immunetics, Cambridge, MA, USA). After fixation of the DNA to the membranes, the membranes were placed in a Miniblotter 45 (Immunetics) with the lanes of DNA at 90° to the lanes of the device. Digoxigenin-labeled whole genomic DNA probes to 41 bacterial species were hybridized in individual lanes of the Miniblotter. After hybridization, the membranes were washed and then incubated with anti-digoxigenin antibody conjugated with alkaline phosphatase. Signals were detected using AttoPhos substrate (Amersham Life Science, Arlington Heights, Illinois, USA) and were read using a Storm Fluorimager (Molecular Dynamics, Sunnyvale, CA, USA), a computer-linked instrument that read the intensity of the fluorescence signals resulting from the probe-target hybridization. Two lanes in each run contained standards at concentrations of 105 and 106 cells of each species. The sensitivity of the assay was adjusted to permit the detection of 104 cells of a given species by adjusting the concentration of each DNA probe. Signals were evaluated using the Storm Fluorimager and converted to absolute counts by comparison with the standards on the same membrane. Failure to detect a signal was recorded as zero.

### Data evaluation

Microbiological data available for each of the 61 subjects were the counts of 41 test species in “supragingival” biofilm samples taken from the dentures and soft tissues of each subject. Counts and proportions of 41 test species were available for one sample per subject from each of the following oral surfaces: tongue dorsum, tongue lateral, tongue ventral, floor of mouth, buccal, hard palate, labial vestibule, attached gingiva, and the exterior surface of the denture hard palate, as well as the counts and proportions of each species in a sample of unstimulated saliva. The counts for each species were averaged per subject before and after denture/soft-tissue cleaning. The percentage of the total DNA probe count was also determined for each species at each site in each subject before and after denture/soft tissue cleaning and then averaged within each subject and across subjects. Significance of differences between pre-cleaning and post-cleaning levels, for each species, was determined using the Friedman test and adjusted for multiple comparisons (14). The mean values for each species were depicted graphically as “microbial profiles” ordered according to the microbial complexes (16).

## RESULTS

**Fig 1:**
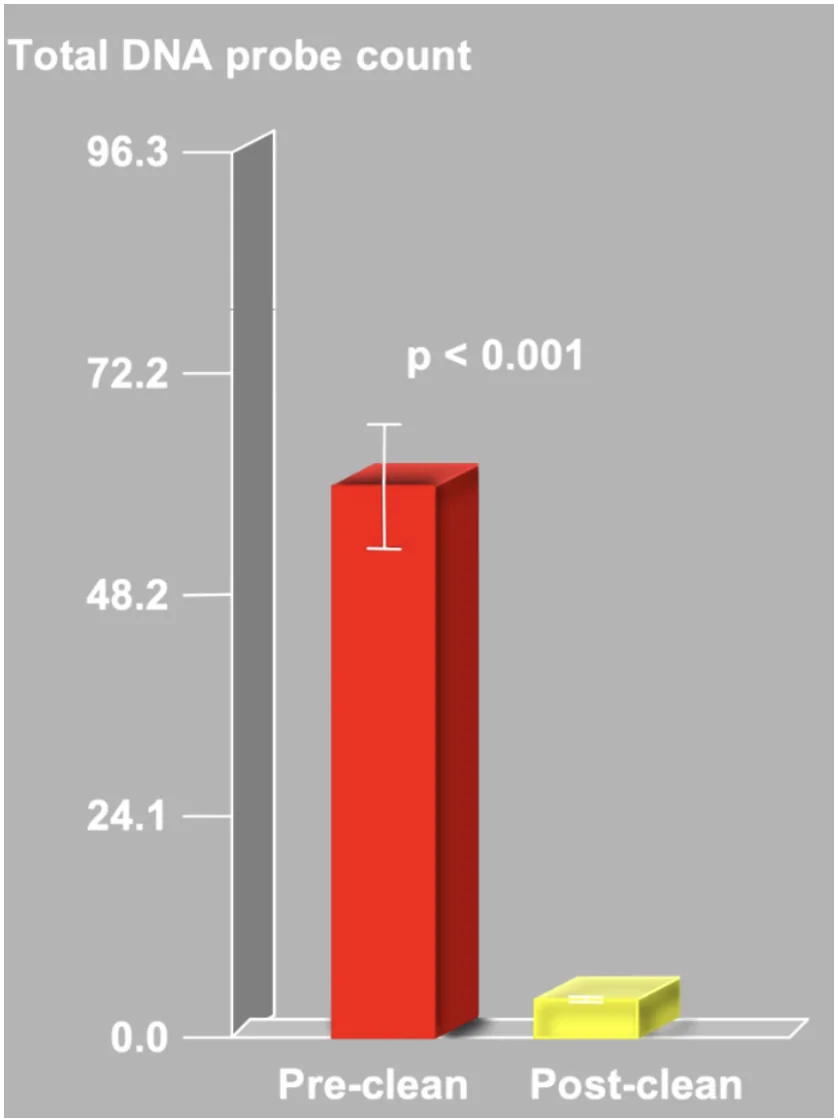
Mean total DNA probe counts (×10^5^, ± SEM) in supragingival plaque samples before and after cleaning in 61 edentulous subjects. A significant decline in overall bacterial load was observed immediately after cleaning, indicating the effectiveness of mechanical disruption on biofilm reduction.

**Fig 2:**
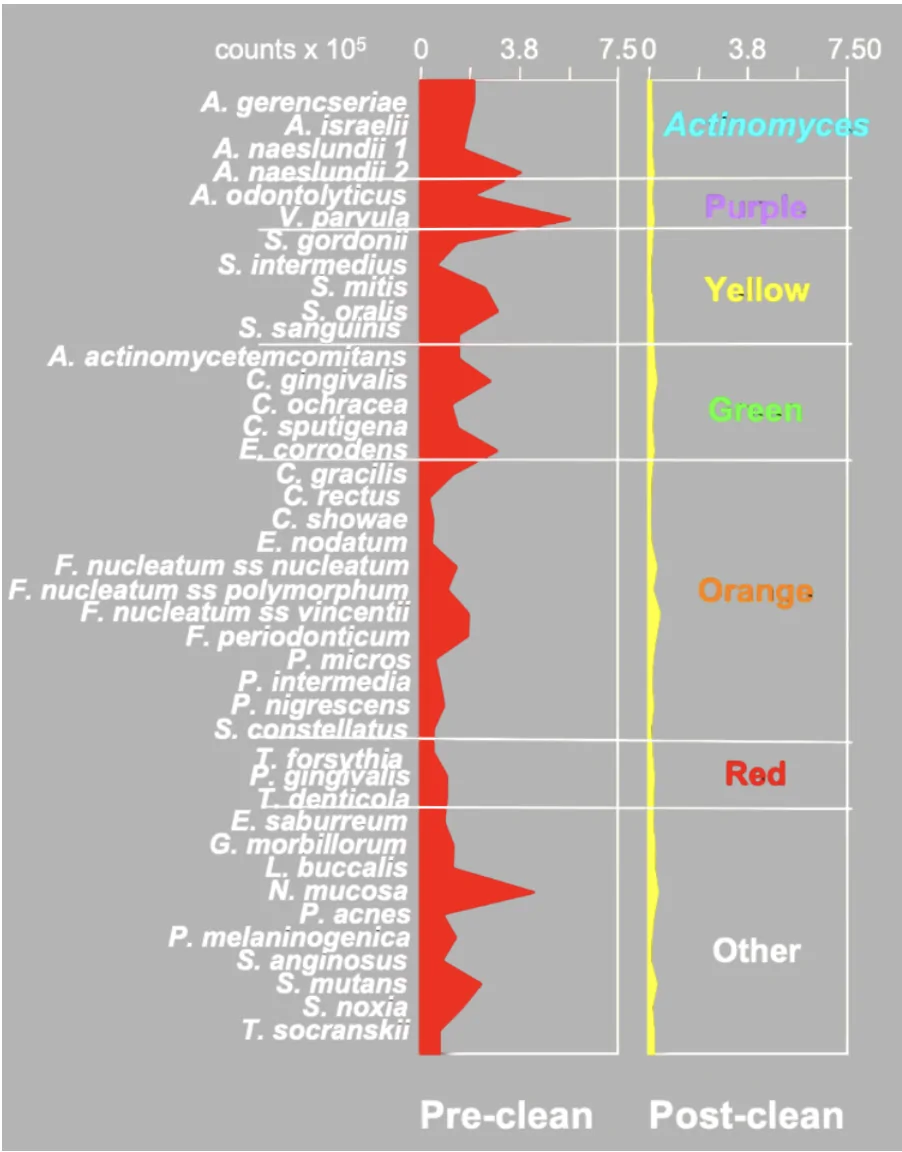
Mean total DNA probe counts (×10^5^, ± SEM) of 41 bacterial species in supragingival plaque samples before and after cleaning in 61 edentulous subjects. Among the 41 species assessed, marked decreases were seen in periodontal pathogens of the red complex and in *Streptococcus mutans*, indicating that cleaning effectively disrupted both periodontal and cariogenic organisms present in the biofilm.

The mean total DNA probe counts for “supragingival” biofilm samples in 61 edentulous subjects before and after cleaning are presented in Fig. 1. The overall bacterial counts decreased significantly immediately after cleaning. The mean counts (×10^5^) for the 41 species before and after cleaning are presented in Fig. 2. It is important to note that periodontal pathogens of the red complex, as well as the caries pathogen, *S. mutans*, that were observed at relatively high proportions in the pre-cleaning biofilm samples, dropped significantly immediately after cleaning.

**Fig 3:**
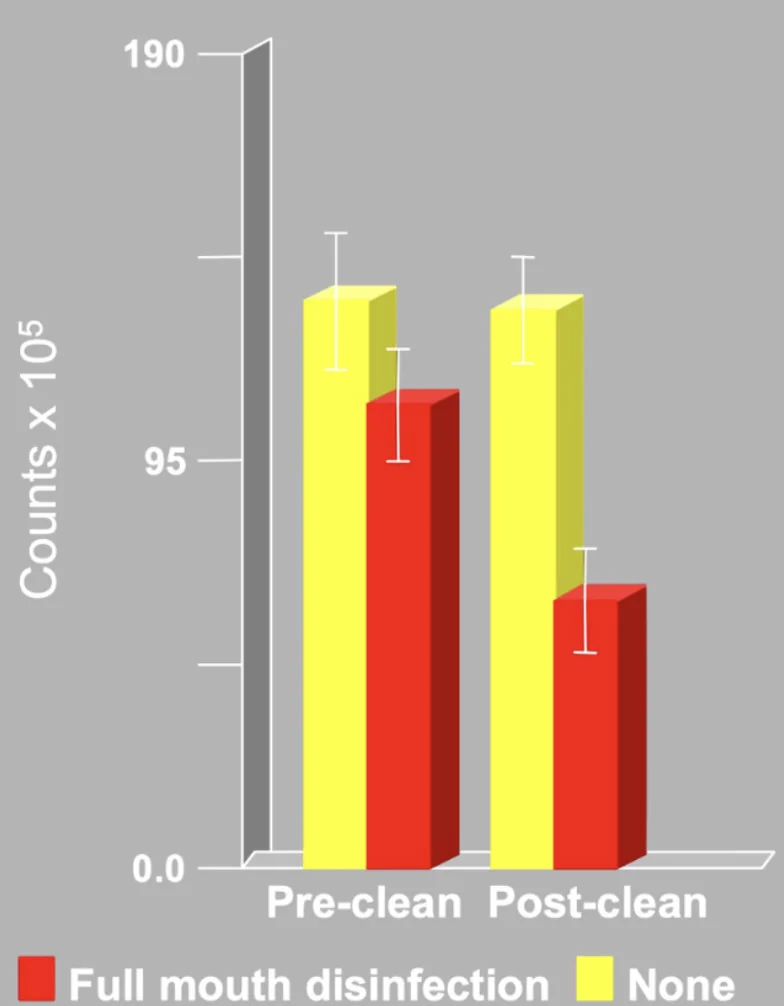
Mean total DNA probe counts (×10^5^, ± SEM) in samples from the tongue dorsum in supragingival plaque samples before and after cleaning in 61 edentulous subjects. Subjects who received chlorhexidine disinfection before sampling showed significantly lower bacterial counts immediately after cleaning compared to those without disinfection, suggesting a transient effect on tongue-associated biofilm.

**Fig 4:**
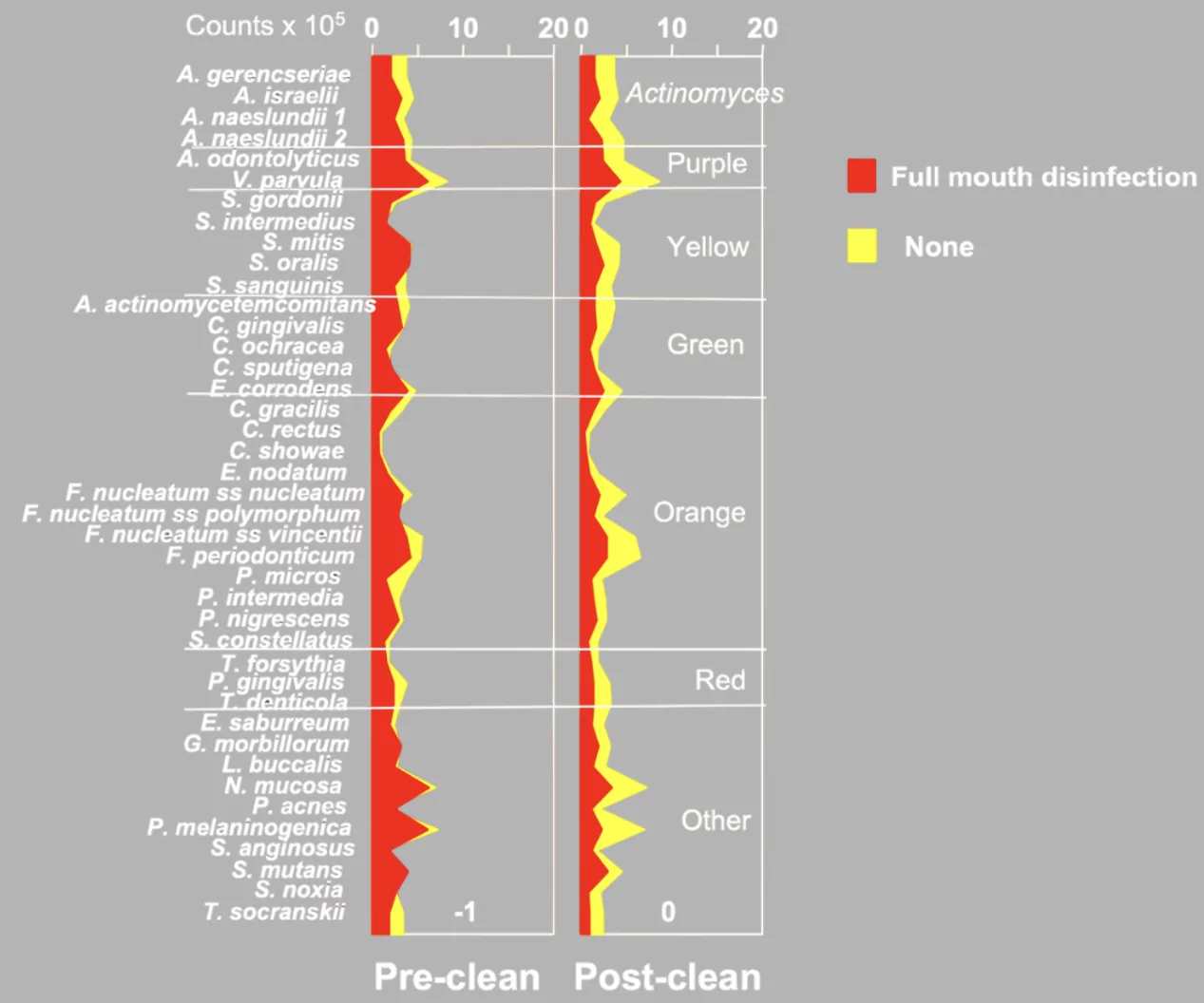
Mean total DNA probe counts (×10^5^, ± SEM) of 41 bacterial species in supragingival plaque samples from the tongue dorsum before and after cleaning in 61 edentulous subjects. Across both groups, the 41 species demonstrated a similar pattern of biofilm re-development following cleaning, with only modest differences observed in those who had received disinfection.

Half of the subjects received soft tissue disinfection with chlorhexidine at the pre-cleaning sampling visit. This one-time soft tissue disinfection had little impact on biofilm re-development on the various biotic soft tissue surfaces, with the exception of the tongue dorsum. Figure 3 presents the mean total DNA probe counts before and after cleaning for tongue dorsum samples from subjects who had received or had not received disinfection. There were significantly lower mean counts immediately after cleaning in subjects who had received the soft tissue disinfection. Examination of the mean counts of the 41 test species before and after cleaning for tongue dorsum samples in the two disinfection groups also showed a similar pattern of plaque re-development, which can be seen in Fig. 4.

## DISCUSSION

This study presented an effective approach for reducing bacterial counts on both dentures and the soft tissues of complete-denture wearers. It specifically examined the effect of disinfecting the dentures together with the oral mucosa compared with disinfecting the dentures alone and found that treating both surfaces produced a greater and more sustained reduction in the microbial burden. Because elderly edentulous individuals represent a significant and steadily increasing portion of the population (50, 51), the authors emphasize that these patients, just as dentate patients do, should visit a dentist or dental hygienist for periodic check-ups and maintenance. Regular professional appointments allow for thorough cleaning of the dentures and the supporting tissues, evaluation of fit and function, and reinforcement of daily hygiene practices that help preserve oral and systemic health.

The disinfection method used in the investigation proved to be highly effective in significantly lowering the counts of oral microbes in the complete-denture-wearing patient. These findings suggest that professional denture disinfection protocols incorporating evaluation and management of oral soft tissues may provide additional microbial reduction compared with denture cleaning alone. Professional denture disinfection markedly reduced microbial biofilm in these individuals, underscoring the critical role of prosthesis hygiene in controlling the overall oral microbial load. Both the test and the control groups showed substantial decreases in bacterial counts, including established periodontal pathogens and cariogenic species such as *S. mutans*, demonstrating that even conventional cleaning offers measurable benefit while combined denture-and-tissue disinfection provides an additional advantage. These findings support previous observations that dentures and oral mucosal surfaces in edentulous individuals can harbor microorganisms traditionally associated with periodontal and cariogenic biofilms, highlighting that tooth loss does not eliminate the presence of potentially pathogenic oral microbial communities. As abiotic surfaces, dentures and oral mucosal surfaces can serve as reservoirs for pathogenic microorganisms implicated not only in oral diseases but also in systemic infections such as aspiration pneumonia and cardiometabolic disorders through pathways involving bacteremia and chronic low-grade inflammation (52–55). In summary, the study reinforces the need for regular professional denture and mucosal disinfection as a key component of preventive care for the growing edentulous population.

Members of the red complex—*P*. *gingivalis*, *T. forsythia*, and *T. denticola*—have been detected on denture surfaces, which serve as persistent abiotic reservoirs for oral biofilms. These organisms are classically associated with periodontitis (16) but have also been identified extraorally, including in the gastrointestinal tract (56, 57). Recent microbiome studies suggest that oral pathogens can translocate to the gut via swallowed saliva and biofilm fragments, where they may contribute to intestinal dysbiosis, low-grade inflammation, and systemic immune modulation (58, 59). Thus, dentures may represent a potential source of continuous microbial exposure to the gastrointestinal tract, although the impact of denture-associated biofilms on gut microbial composition remains unclear.

Although strong studies directly quantifying red complex bacteria on denture surfaces before and after cleaning are lacking, related work demonstrates that professional denture cleaning protocols (e.g., hypochlorite, chlorhexidine, microwave disinfection, and photodynamic therapy) reduce total bacterial load, gram-negative anaerobes, and *Candida* species on dentures (60–63). These reductions suggest that effective disinfection may also diminish reservoirs of red complex organisms, thereby potentially limiting their contribution to the oral–gut microbial axis and systemic health outcomes.

The tongue is a major contributor to high loads of microorganisms, and rapid recolonization is likely. The additional benefit of broader soft tissue disinfection with chlorhexidine was evident primarily on the tongue (64, 65), where microbial load is typically high and colonization can rapidly recolonize dental prostheses (66). This finding underscores the dynamic interaction between abiotic denture surfaces and biotic oral tissues, both of which contribute to the oral microbial ecosystem and, potentially, to microbial spillover into the gastrointestinal tract. However, the limited effect at other sites indicates that soft tissue disinfection may need to be targeted or repeated more frequently to achieve broader microbial suppression.

Historically, culture-based methods underestimated the diversity of organisms present on dentures. The DNA–DNA hybridization (checkerboard hybridization) (48) allowed simultaneous detection of multiple species at higher sensitivity, demonstrating that denture biofilms host a wide range of organisms also implicated in periodontal and systemic conditions. More recently, high-throughput sequencing and qPCR approaches, such as 16s and shotgun, have expanded upon these earlier findings, providing greater taxonomic resolution and quantitative insights into the denture microbiome (67). Microbiome studies further suggest that oral pathogens from these reservoirs can translocate to the gastrointestinal tract via swallowed saliva and biofilm fragments, contributing to intestinal dysbiosis, low-grade inflammation, and systemic immune modulation. These findings highlight a potential area of investigation regarding whether denture-associated microbial reservoirs influence broader host–microbiome interactions.

From a clinical perspective, these results highlight the importance of integrating both prosthesis and soft tissue management in oral care protocols for denture wearers. Denture disinfection should remain the cornerstone of care, but adjunctive measures such as periodic tongue and mucosal decontamination may provide incremental benefits, particularly for patients at higher risk of systemic complications. These findings are consistent with the concept that oral health maintenance in elderly and medically compromised populations requires attention not only to oral disease prevention but also to systemic health through the modulation of oral and gut microbiota.

Some limitations should be noted. The study duration was relatively short, and long-term outcomes related to recolonization patterns and clinical endpoints, including mucosal inflammation, denture stomatitis, gut microbial changes, or systemic health outcomes, were not assessed. Therefore, the immediate post-treatment microbiological findings cannot establish the long-term durability of microbial reductions or their sustained clinical implications. Additionally, this historical randomized clinical trial was conducted without an a priori sample size calculation, and future prospective studies may benefit from contemporary longitudinal analytical approaches, such as mixed-effects models, to further evaluate intergroup differences over time. Furthermore, chlorhexidine, while effective, has known side effects such as taste alteration and staining that may limit long-term compliance. Future studies should investigate alternative antimicrobial agents, optimized delivery systems, or patient-friendly regimens that sustain microbial reductions on abiotic and biotic surfaces while also exploring their downstream effects on the gut microbiome.

This study reinforces that professional denture disinfection effectively lowers the oral microbial load on abiotic prosthetic surfaces, including red complex pathogens with potential links to gut dysbiosis. Adjunctive soft tissue disinfection provides additional site-specific benefit. A combined approach may therefore represent a more comprehensive strategy for controlling oral biofilm and mitigating risks associated with the oral–gut microbial axis in the denture-wearing population.

### Conclusion

This study demonstrates that professional denture disinfection significantly reduces microbial biofilm in complete denture patients, effectively lowering overall bacterial load as well as specific pathogens such as members of the red complex and *S. mutans*. While adjunctive soft tissue disinfection with chlorhexidine produced an additional short-term reduction in tongue dorsum biofilm, its effect on other oral surfaces was limited. These findings highlight that denture disinfection alone is highly effective, but targeted soft tissue treatment may offer added benefits at specific sites prone to biofilm accumulation, given the well-established links between oral pathogens and systemic health in the edentulous population. The findings support the potential value of professionally guided denture and soft tissue hygiene protocols in reducing oral microbial burden among complete-denture wearers. Further longitudinal studies are needed to determine whether these microbiological improvements translate into sustained clinical or systemic benefits. Longitudinal studies with longer follow-up are needed to determine the sustained impact of combined disinfection strategies on microbial recolonization and patient-centered outcomes.

## Data Availability

The data supporting the findings of this study are available from the corresponding author upon reasonable request. This investigation represents the second phase of a previously published study, which can be referenced at: DOI: 10.1111/j.1532-849X.2008.00301.x.
